# Free Fatty Acids and Free Fatty Acid Receptors: Role in Regulating Arterial Function

**DOI:** 10.3390/ijms25147853

**Published:** 2024-07-18

**Authors:** Fengzhi Yu, Boyi Zong, Lili Ji, Peng Sun, Dandan Jia, Ru Wang

**Affiliations:** 1 School of Exercise and Health, Shanghai University of Sport, Shanghai 200438, China; yu19960701@126.com (F.Y.); 2311516011@sus.edu.cn (L.J.); 2 College of Physical Education and Health, East China Normal University, Shanghai 200241, China; boyi0303@126.com (B.Z.); psun@tyxx.ecnu.edu.cn (P.S.); 3 Key Laboratory of Adolescent Health Assessment and Exercise Intervention of Ministry of Education, East China Normal University, Shanghai 200241, China

**Keywords:** free fatty acids, free fatty acid receptors, G protein-coupled receptors, arterial function

## Abstract

The metabolic network’s primary sources of free fatty acids (FFAs) are long- and medium-chain fatty acids of triglyceride origin and short-chain fatty acids produced by intestinal microorganisms through dietary fibre fermentation. Recent studies have demonstrated that FFAs not only serve as an energy source for the body’s metabolism but also participate in regulating arterial function. Excess FFAs have been shown to lead to endothelial dysfunction, vascular hypertrophy, and vessel wall stiffness, which are important triggers of arterial hypertension and atherosclerosis. Nevertheless, free fatty acid receptors (FFARs) are involved in the regulation of arterial functions, including the proliferation, differentiation, migration, apoptosis, inflammation, and angiogenesis of vascular endothelial cells (VECs) and vascular smooth muscle cells (VSMCs). They actively regulate hypertension, endothelial dysfunction, and atherosclerosis. The objective of this review is to examine the roles and heterogeneity of FFAs and FFARs in the regulation of arterial function, with a view to identifying the points of intersection between their actions and providing new insights into the prevention and treatment of diseases associated with arterial dysfunction, as well as the development of targeted drugs.

## 1. Introduction

Cardiovascular diseases (CVDs) are the primary cause of death globally [1,2]. A review of research data indicates that CVDs affect 523 million people globally, with 18.6 million deaths attributed to CVDs in 2019 [3]. CVDs are responsible for approximately 50% of all deaths in developed countries, with the incidence of this disease increasing annually in developing countries [4]. Atherosclerosis is the primary cause of CVD mortality, accounting for 84.9% of all CVD-related deaths [5]. Arterial stiffness and endothelial dysfunction represent the most prevalent forms of CVDs, predominantly resulting from dysfunction of VECs and lipid metabolism. The primary symptoms include vascular stenosis, vascular calcification, thickening of the vessel wall, formation of atheromatous plaques, and plaque rupture, which are the main causes of acute CVDs such as coronary heart disease, cerebral infarction, and cerebral thrombosis [6,7,8]. Serum FFAs have been identified as an independent risk factor for atherosclerosis and arterial dysfunction [9,10,11].

FFAs are physiological ligands for specific G protein-coupled receptors (GPCRs). They serve as an important energy source for human tissues and are involved in the regulation of cellular function, receptor signalling, gene expression, and whole-body energy homeostasis under physiological conditions [12]. The accumulation of saturated FFAs represents a significant contributing factor to lipotoxicity, which in turn results in the dysfunction and apoptosis of VECs [13]. FFAs, also known as non-esterified fatty acids (NEFAs), are independent risk factors for CVDs and are involved in the pathological process of CVDs [14]. Studies have revealed that an excess of fatty acids (FAs) can contribute to the generation of a remnant-rich dyslipidemia and the preconditioning of the arterial intima for lipoprotein deposition via alterations in the expression of matrix proteoglycans. Normalizing FA levels should be a primary objective in the treatment of atherogenic dyslipidemia associated with insulin resistance (IR) [15]. FFARs are receptors for FFAs, including GPR40 (FFAR1), GPR120 (FFAR4), GPR41 (FFAR2), and GPR43 (FFAR3). They are expressed throughout the body and regulate a multitude of biological processes. The activation of FFARs plays a pivotal role in regulating various physiological processes, such as insulin secretion from pancreatic β-cells, adipocyte differentiation, enteroendocrine cells, enteroinsulin secretion, regulation of food intake, adipose tissue biology, inflammation, and neuronal activity [16,17,18,19,20,21,22,23,24]. FFARs play a crucial role in the pathogenesis of various diseases, including diabetes [25,26,27,28], obesity, metabolic syndrome [29,30,31,32], asthma [33], arthritis [34], inflammatory bowel disease [35], and CVDs (arrhythmias, heart failure, hypertension, and myocardial infarction) [36,37,38,39]. A number of agonists of FFARs have been developed and tested in animal models and human trials [40,41,42,43]. Nevertheless, the relevance of FFARs in arterial function has not yet been comprehensively addressed. The objective of this paper is to conduct a comprehensive review of existing studies with the aim of exploring the regulatory role of FFAs and FFARs in arterial function.

## 2. FFAs and FFARs

### 2.1. The Classification and Production Pathways of FFAs

FAs are carboxylic acids with long saturated or unsaturated chains. The majority of naturally occurring FAs comprise a straight chain and 4–28 even carbon atoms [44]. FAs can be classified into three categories based on the length of the chain: short-chain fatty acids (SCFAs), medium-chain fatty acids (MCFAs), and long-chain fatty acids (LCFAs) [45] (see Table 1). It should be noted that SCFAs contain six or fewer carbon atoms, while MCFAs contain 6–12 carbon atoms, and LCFAs contain 12 or more carbon atoms [46]. The SCFAs include acetic acid (C2:0), propionic acid (C3:0), butyric acid (C4:0), valeric acid (C5:0), and hexanoic acid (C6:0). In contrast, the MCFAs comprise caprylic acid (C8:0), decanoic acid (C10:0), and lauric acid (C12:0), as well as other saturated fatty acids (SFAs), such as myristic acid (C14:0), palmitic acid (PA) (C16:0), stearic acid (SA) (C18:0), and all types of unsaturated FAs, collectively referred to as LCFAs [22]. FAs can be classified based on the presence of double bonds. SFAs lack double bonds, monounsaturated fatty acids (MUFAs) have only one double bond, and polyunsaturated fatty acids (PUFAs) contain two or more double bonds [47]. Carbon-carbon double-bonded unsaturated fatty acids (UFAs) comprise both cis and trans isomers, with the cis isomer being the predominant configuration of most natural UFAs [48]. FAs are primarily found in adipose tissue and cellular membranes and are components of phospholipids and glycolipids. FFAs are the form in which FAs leave the cell for translocation to other parts of the body for use, usually as NEFAs [49,50]. The geometric differences between various types of SFAs and UFAs play a crucial role in various biological processes and the formation of biological structures, such as cell membranes. It is typical for FFAs to be bound to transport proteins, such as albumin [44,51].

### 2.2. The Classification and Associated Signalling Pathways of FFARs

**Figure 1:**
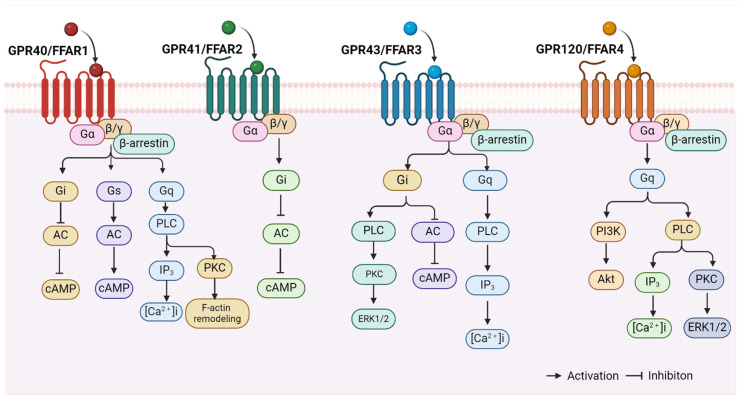
FFAR-related signalling pathways. FFARs, including GPR40 (FFAR1), GPR43 (FFAR2), GPR41 (FFAR3), and GPR120 (FFAR4), bind to intracellular heterotrimeric G proteins and β-arrestin proteins to initiate intracellular signalling cascades. The figure is created with BioRender. Abbreviations: AC, adenylate cyclase; cAMP, cyclic adenosine monophosphate; PLC, phospholipase C; IP3, inositol triphosphate; [Ca^2+^]i, intracellular free Ca^2+^ concentration; PKC, protein kinase C; ERK1/2, extracellular regulatory protein kinase1/2; PI3K, phosphatidylinositol-3-hydroxykinase; Akt/PKB, protein kinase B.

The available evidence indicates that FFARs include GPR40, GPR43, GPR41, and GPR120 [24]. These receptors belong to the retinoid-like family of GPCRs, and each contains seven transmembrane structural domains [45,65,66]. It has been demonstrated that FFARs bind to intracellular proteins, such as heterotrimeric G proteins and β-arrestin, to initiate intracellular signalling cascades [67,68,69] (see Figure 1). GPR40 and GPR120 were activated by LCFAs such as palmitate, oleate, and linoleate, whereas GPR43 and GPR41 were mainly activated by SCFAs (e.g., acetate, butyrate, and propionate) [21,70,71,72]. Currently, only the structure of GPR40 has been revealed by crystallographic methods [73]. In humans, the genes encoding the first three members of the FFAR family are clustered in tandem on the long arm of chromosome 19 (19q13.1) and precede the CD22 gene alignment [74]. The amino acid sequence similarity between GPR40 and GPR41 is 34%, between GPR40 and GPR43 is 41%, and between GPR43 and GPR41 is 52% [74]. The GPR120 gene, located on the long arm of chromosome 10 (10q23.33), exhibits a lower degree of similarity to GPR40 and is known to express both long (BC101175, GPR120L) and short (NM-181745, GPR120S) isoforms in humans [75]. GPR40 and GPR120 can be activated by MCFAs and LCFAs, whereas GPR41 and GPR43 are activated by SCFAs [76,77]. The half-maximal effective concentration (EC50) values for SFCAs are between 0.1 and 1.0 mM for GPR43 and GPR41, while those for LCFAs are 1.0 to 30 μM for GPR40 and GPR120 [78]. In recent times, there has been a great deal of interest in the role of FFARs in the regulation of the cardiovascular system, with a particular focus on the regulation of arterial function.

## 3. Modulation of Arterial Function by FFAs

Dietary FAs serve a multitude of functions within the body. They provide energy, participate in cellular signalling cascades, and function as components of various molecules. Additionally, FAs play a crucial role in mediating inflammation, and maintaining the balance of FA intake is of paramount importance for the preservation of cellular function and tissue homeostasis [79]. A substantial body of research has indicated that diets with excessive amounts of SFAs, high ratios of pro-inflammatory omega-6 PUFAs may result in diet-induced obesity and various obesity-related comorbidities, including type 2 diabetes mellitus, CVDs, and musculoskeletal disorders [80,81,82]. An excessive intake of SFAs represents a significant contributing factor to the development of atherosclerosis. This is due to their ability to stimulate the production of low-density lipoprotein (LDL), which is regarded as the primary risk factor for atherosclerosis [83]. Research has demonstrated that LCFAs increase the risk of coronary heart disease more than SCFAs and MCFAs. Furthermore, excess FFAs exert a detrimental effect on the functionality of VECs, macrophages, and VSMCs in the vessel wall, which in turn impairs arterial function [84]. Consequently, elevated plasma FFA levels represent a significant contributing factor to arterial dysfunction. The regulation of several aspects of arterial function is mediated by FFAs, including endothelial dysfunction, angiogenesis, hypertension, and atherosclerosis.

### 3.1. Effects of FFAs on Endothelial Dysfunction

Excessive levels of UFAs, such as oleic acid (OA) and linoleic acid (LA), and SFAs, such as PA, have been demonstrated to induce endothelial dysfunction through a number of mechanisms. These include impaired insulin signalling, dysregulated nitric oxide (NO) synthesis, inflammation, activation of the renin-angiotensin system (RAS), and apoptosis. The primary causes of endothelial dysfunction induced by FFAs are inflammation, oxidative stress (OS), and IR [85,86,87]. NO is a vasodilatory factor, and the release of NO induced by changes in intracellular calcium levels plays an important role in the mechanosensitive response of VECs [88]. Free UFAs, such as OA and LA, have been demonstrated to reduce intracellular calcium mobilisation and influx in bovine aortic endothelial cell (BAEC) cultures. This impairment of NO production and release could potentially affect arterial diastole [89]. It has been demonstrated that SFAs can induce injury to VECs and increase the risk of CVDs by promoting atherosclerosis. In contrast, PUFAs, such as docosahexaenoic acid (DHA), are thought to inhibit the injury to VECs that is induced in the early stages of CVDs. Eicosapentaenoic acid (EPA) has been shown to prevent vascular endothelial dysfunction induced by SFAs [11]. Nevertheless, elevated levels of UFAs may also induce apoptosis of VECs through the activation of protein phosphatase 2Cb (PP2Cb) [90]. Ishida and colleagues [91] discovered that human umbilical-vein endothelial cells (HUVECs) stimulated by PA (100 μM) exhibited increased expression of intracellular adhesion molecules, cytokines, and inflammatory factors, including ICAM-1, MCP-1, IL-6, and PTX3. Furthermore, PA stimulates the expression of long-chain acyl-CoA synthetase and the cell cycle protein-dependent kinase inhibitor p21, as well as the phosphorylation of p65, which collectively promote inflammatory responses.

**Figure 2:**
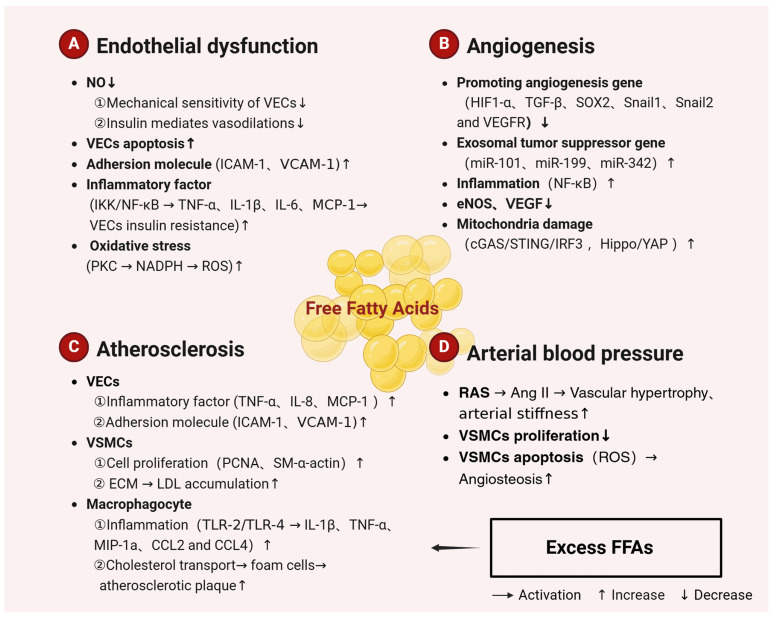
Mechanisms related to the regulation of arterial function by FFAs. Excessive dietary intake of SFAs is a major cause of atherosclerosis. SFAs trigger endothelial dysfunction, reduce angiogenesis and atherosclerosis, and increase arterial blood pressure, leading to arterial dysfunction. Most UFAs, on the other hand, are protective against CVDs. The figure is created with BioRender. Abbreviations: NO, nitric oxide; VECs, vascular endothelial cells; VSMCs, vascular smooth muscle cells; ICAM-1, intercellular adhesion molecule-1; VCAM-1, vascular cell adhesion molecule-1; IKK, I-kappa-B kinase; NF-κB, nuclear factor kappa B; TNF-α, tumor necrosis factor-α; TNF-β, tumor necrosis factor β; IL, interleukin; MCP-1, monocyte chemoattractant protein-1; NADPH, nicotinamide adenine dinucleotide phosphate; ROS, reactive oxygen species; PCNA, proliferating cell nuclear antigen; ECM, extracellular matrix; TLR-2, Toll-like receptor-2; TLR-4, Toll-like receptor-4; MIP-1α, macrophage inflammatory protein-1α; CCL2, chemokine (C-C motif) ligand 2; CCL4, chemokine (C-C motif) ligand 4; HIF-1α, hypoxia-inducing factor 1α; SOX2, sex determination region Y box protein 2; VEGFR, vascular endothelial growth-factor receptor; cGAS, cyclic-bird adenylate synthetase; STING, stimulator of interferon genes; IRF3, interferon regulatory factor 3; YAP, Yes-associated protein; RAS, renin-angiotensin system; AngII, angiotensin II; SFAs, saturated fatty acids.

It has been demonstrated that excess FFAs represent a significant source of reactive oxygen species (ROS) in VECs, which can subsequently lead to OS events. These effects were observed at high glucose concentrations [92]. The production of ROS is primarily attributed to the activation of NADPH oxidase by PKC [93]. Additionally, PKC exerts a role in FFA-induced inflammation. Elevated plasma FFA levels have been linked to IKK/nuclear factor kappa B (NF-κB) inflammatory signalling, which can result in the activation of TNF-α, IL1-β, and IL-6, as well as increased MCP-1 plasma levels [94]. These components contribute to the development of chronic inflammation and may result in IR in VECs [16,95]. Another study has demonstrated that elevated FFA levels lead to OS, which impairs vasodilatory NO production and insulin-mediated vasodilation [96]. The administration of 5-aminoimidazole-4-carboxamide riboside has been demonstrated to protect VECs from oxidative damage induced by chronic palmitate overdose [97]. Moreover, physiological doses of oleic and palmitic acids can also protect VECs from OS. The pretreatment and co-treatment of cells with physiological concentrations of PA or OA in the low micromolar range effectively protect cell viability from oxidative damage [98] (see Figure 2). It can be seen from the above that high concentrations of FFAs in the vasculature have the effect of triggering arterial dysfunction. This is achieved by promoting the secretion of adhesion molecules, cytokines, and inflammatory factors; cholesterol transport; and by increasing the levels of OS in VECs. This, in turn, leads to the following consequences: endothelial oxidative damage and endothelial dysfunction. Conversely, physiological doses of FFAs afford VECs protection from OS.

### 3.2. Effects of FFAs on Angiogenesis

Metabolic processes, including glycolysis, fatty acid oxidation, and glutamine metabolism, play distinctive and pivotal roles in angiogenesis [99]. Elevated free fatty acid levels have been demonstrated to cause metabolic stress, which has been shown to inhibit endothelial angiogenesis [100]. DHA, an omega-3 free fatty acid, has anti-angiogenic effects. Human breast cancer cell lines, including MDA-MB-231 and BT-474, were treated with 100 μM DHA for 24 h. In both normoxic and hypoxic conditions, DHA treatment resulted in a significant reduction in the expression of pro-angiogenic genes, including HIF1-α, TGF-β, SOX2, Snail1, Snail2, and VEGFR. Additionally, the expression levels of tumor suppressor microRNAs (miRs), including miR-101, miR-199, and miR-342, were increased, while the expression levels of oncomiRs, including miR-382 and miR-21, were decreased. DHA has the ability to modify the expression and miRs content of pro-angiogenic genes in breast cancer cells and their derived exosomes, thereby inhibiting angiogenesis [101].

In 2023, Wu et al. [102] conducted a study which found that treatment with caffeic acid phenethyl ester (CAPE) significantly inhibited the proliferation, migration, invasion, and angiogenesis of breast cancer MDA-MB-231 cells in the lipopolysaccharide (LPS)-stimulated inflammatory microenvironment, resulting in a decrease in mitochondrial membrane potential. Nevertheless, a study was conducted on the co-culturing of human adipocytes (SW872) cells and human primary myometrial smooth-muscle tumour cells. The study demonstrated that FFAs were transferred from adipocytes to smooth-muscle tumour cells. Additionally, adipocyte-smooth-muscle tumour cell interactions resulted in elevated levels of phosphorylated NF-κB. The phosphorylation of NF-κB plays a key role in the regulation of inflammation, the reorganization of metabolic pathways, and angiogenesis [103]. The Akt, eNOS, and ERK activation responses of 100 µM PA-pretreated HUVECs were markedly diminished in response to VEGF. PA inhibited VEGF-induced angiogenic cord formation in Matrigel [104]. Yuan et al. [100] reported that high concentrations of PA (0.3–0.5 mM) dysregulate the Hippo-YAP pathway, inhibit angiogenesis, and induce mitochondrial damage. Additionally, the researchers discovered that the cytoplasmic DNA sensor cGAS-STING-IRF3 signalling mechanism is activated. Higher levels of circulating OA (400 μM) have been shown to promote angiogenesis and tissue remodelling [105]. Nevertheless, elevated levels of oleic acid have also been associated with the induction of inflammatory and apoptotic processes. Joyal et al. [106] discovered that exposing human retinal pigment epithelial (RPE) ARPE-19 cells to monounsaturated OA at doses greater than 500 μM significantly increased autophagic flux and cell migration. Concurrently, OA treatment stimulated not only AMPK/mTOR/p70S6K signalling but also induced hyperphosphorylation of MAPK pathway mediators, including ERK, JNK, and p38 MAPK, as well as NF-κB activation. Kinase inhibition assays showed that the blockade of the PI3K/Akt, MAPK, and NF-κB pathways prevented the OA-upregulated VEGF transcription and its peptide release (see Figure 2). In conclusion, it appears that metabolic stress induced by elevated, excessive levels of UFAs, such as OA, and SFAs, such as PA, consistently has a negative effect on angiogenesis. Nevertheless, further research is required to fully elucidate the specific evidence and mechanisms involved.

### 3.3. Effects of FFAs on Atherosclerosis

Elevated plasma FFAs have been linked to a number of conditions, such as atherosclerosis, pancreatic β-cell dysfunction, metabolic syndrome, and IR [107,108,109]. In a study of a multiethnic cohort without baseline CVDs, the relationship between circulating omega-3 and omega-6 poly UFAs and arterial elasticity was examined. The study found that circulating omega-3 and omega-6 PUFAs were associated with large arterial elasticity (LAE) and small arterial elasticity (SAE) in participants of the Multi-Ethnic Study of Atherosclerosis (MESA) [110]. Another study demonstrated that a high-fat, high-cholesterol diet does not induce atherosclerosis in mice with an inactivated Δ6-fatty acid desaturase, which is deficient in omega-3 and omega-6 PUFAs synthesis [111]. FFAs have been shown to contribute to the development of atherosclerosis by affecting the function of VECs, macrophages, and VSMCs [112,113,114].

In VECs, elevated levels of FFAs promote the levels of inflammatory factors such as TNF-α, MCP-1, and IL-8 and increase the secretion of VCAM-1 and ICAM-1 [113]. In a study of VSMCs, Li et al. [115] observed that trilinolein and triolein increased the expression of PCNA and MCP-1 protein and mRNA in human umbilical-vein smooth-muscle cells (HUVSMCs), while concomitantly decreasing SM-α-actin expression. The results demonstrated that tristearin, trilinolein, and triolein significantly promote low-density lipoprotein (ox-LDL)-induced proliferation of HUVSMCs and inhibited atherogenesis. Additionally, it has been demonstrated that elevated levels of FFAs in VSMCs result in a reduction in the production of extracellular matrix, which in turn leads to an increase in LDL accumulation [9,84]. Furthermore, persistent excess of FFAs and glucose in plasma has been identified as a significant contributor to the development of metabolic inflammation and damage-associated molecular patterns (DAMPs) [116,117]. Excessive plasma FFAs induce the production of IL-1β, TNF-α, MIP-1α, CCL2, and CCL4 in macrophages via TRL-2 or TLR-4, thereby triggering arterial inflammation and inducing atherogenesis [118,119,120,121,122]. FFAs are involved in the transformation of macrophages into foam cells by affecting cholesterol transport [113] (see Figure 2). In conclusion, high levels of FFAs play a crucial role in atherosclerosis by regulating inflammation levels, adhesion molecules, cell proliferation, and the extracellular matrix in VECs, VSMCs, and macrophages.

### 3.4. Effects of FFAs on Arterial Blood Pressure

A number of prospective studies have indicated that elevated serum-FFA concentrations are an independent risk factor for the development of arterial hypertension [96]. The renin-angiotensin system (RAS) plays a crucial role in regulating arterial blood pressure. Angiotensin II (Ang II) has been identified as a potent vasoconstrictor [123]. Ang II has been demonstrated to suppress the oxidative pathway of FAs, which have been shown to activate the RAS [124]. The gene expression of enzymes involved in the breakdown of fats and the oxidation of FAs is elevated in mice lacking in angiotensin-converting enzyme (ACE^−/−^), which stimulates the generation of FFAs [125]. The release of FFAs induced by the activation of LPL was found to elevate blood pressure [96]. Furthermore, elevated levels of FFAs have been demonstrated to increase OS, resulting in a reduction in the production of vasodilatory NO and impaired insulin-mediated vasodilation. Furthermore, excess FFAs can also stimulate the proliferation of VSMCs, which in turn leads to vascular hypertrophy and stiffness of the vascular wall. Another study demonstrated that high concentrations of PA (100 μM) mediate apoptosis in VSMCs through the activation of the TLR4 pathway and the induction of ROS generation [126]. High-fat diet-caused vascular calcification was associated with PA-induced downregulation of SIRT6. The study found that overexpression of SIRT6 reduced palmitate-induced calcification and apoptosis in VSMCs. Primary VSMCs treated with high concentrations of PA showed decreased expression of SIRT6 and increased expression of BMP2 and RUNX2, resulting in apoptosis and vascular calcification [127] (see Figure 2). This evidence suggests that excess FFAs primarily stimulate the proliferation, migration, apoptosis, and vascular calcification of VSMCs. This leads to vascular hypertrophy and vessel-wall stiffness, which are associated with the development of arterial hypertension.

In conclusion, it is believed that a range of FFAs, such as PUFAs, at normal physiological concentrations, prevent the onset of impairments to arterial function, such as vascular endothelial dysfunction. Nevertheless, elevated levels of FFAs above those considered normal are the primary cause of induced arterial dysfunction. High concentrations of FFAs in the vasculature regulate inflammatory levels, adhesion molecule and cytokine secretion, cholesterol transport, cell proliferation and extracellular matrix formation in VECs, VSMCs, and macrophages. This results in endothelial dysfunction, vascular hypertrophy, and vascular wall stiffness, thereby promoting the development of arterial hypertension and atherosclerosis. Furthermore, it was demonstrated that metabolic stress induced by elevated serum concentrations of FFAs significantly inhibits angiogenesis. Nevertheless, research on the adverse effects of elevated concentrations of FFAs on arterial function has primarily focused on the relationship between FFAs and arterial function, with limited investigation into the intermediate mechanisms. For example, it is unclear whether FFAs exert deleterious effects on arteries through binding to FFARs. Further investigation of these mechanisms is recommended in subsequent studies.

## 4. Modulation of Arterial Function by FFARs

### 4.1. GPR40/FFAR1

GPR40 is primarily distributed in VECs, nerves, pancreatic β-cells, enteroendocrine cells, and immune cells [18,19,20,40,128,129,130]. It was demonstrated that GPR40, which is primarily activated by MCFAs and LCFAs, can couple with Gq proteins to initiate downstream PLC activation and promote IP3 or diacylglycerol-induced phosphorylation of PKC, resulting in elevated intracellular Ca^2+^ levels [131,132]. Consequently, in numerous in vitro studies, the extent of GPR40 activation was quantified based on the intracellular concentration of Ca^2+^. Additionally, it has been reported that GPR40 can be coupled to Gi and Gs proteins to either inhibit or promote cAMP production by activating AC [133,134,135]. GPR40 signalling is associated with the recruitment of β-arrestin-1 and 2 [136,137]. The coupling of GPR40-Gq activates the ERK1/2 signalling pathway [18,138], which in turn promotes cellular Ca^2+^ release and ERK1/2 phosphorylation [21].

In the vasculature, GPR40 is a low-affinity receptor for EETs. The epoxidation of EETs by arachidonic acid enhances the agonist activity of GPR40. Activation of GPR40 by 11,12-EET has been demonstrated to increase the expression of Cx43 and COX-2 in VECs, with the promotion of ERK phosphorylation being a key mechanism involved in this process [139]. In a study by Park et al. [140], it was discovered that GPR40 was involved in the secretion of IL-6 in ECs stimulated by PA (100 or 250 μM). Additionally, PA exerts a synergistic effect on LPS-induced IL-6 expression in VECs, which is mediated by MAPK and NF-κB-related signalling pathways. In HUVECs, AM1638, a GPR40 full agonist, enhanced nuclear factor erythroid 2-related factor 2 (NRF2) translocation to the nucleus and heme oxygenase-1 (HO-1) expression. This resulted in the blockade of PA-induced superoxide production and the enhancement of HUVECs viability [141].

**Figure 3:**
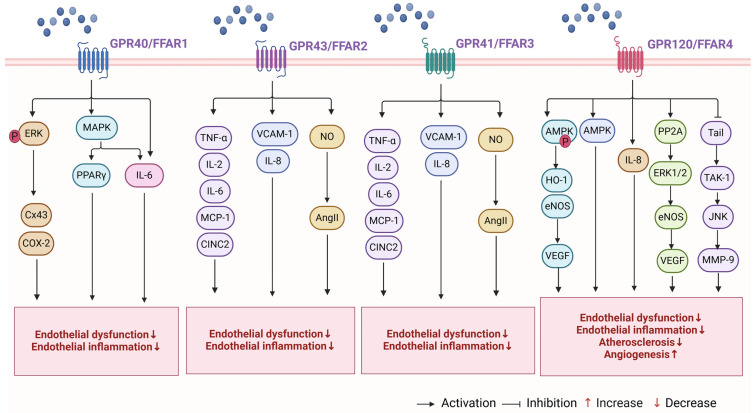
Mechanisms involved in the regulation of arterial function by FFARs. FFARs bind to arterial FFARs and regulate arterial functions, including endothelial dysfunction, inflammation, angiogenesis, atherosclerosis, and arterial blood pressure. The figure is created with BioRender. Abbreviations: ERK, extracellular signal-regulated kinase; Cx43, gap connexin 43; COX-2, cyclooxygenase-2; MAPK, mitogen-activated protein kinase; PPARγ, peroxisome proliferator-activated receptor γ; AMPK, AMP-activated protein kinase; CREB, cAMP-response element binding protein; PGC1, peroxisome bioactivator receptor gamma coactivator 1; CINC2, cytokine-induced neutrophil chemoattractant 2; HO-1, heme oxygenase-1; PP2A, protein phosphatase 2A; eNOS, endothelial nitric oxide synthase; TAK-1, transforming growth factor-beta activating protein 1; MMP-9, matrix metalloproteinase 9; JNK, c-Jun amino-terminal kinase; P, phosphorylation.

Rosiglitazone (RGZ) has been demonstrated to promote the transcription of genes associated with human VECs through the activation of the GPR40/p38 MAPK-related pathway and PPARγ. The induction of PPARγ target genes by RGZ in primary human pulmonary artery endothelial cells was inhibited by knockdown of p38 MAPK or GPR40 [142]. The presence of excessive lipids in the bloodstream was found to impede retinal autophagy, which in turn led to the development of pathological angiogenesis in the Vldlr^−/−^ RAP model. Additionally, the FAs derived from triglycerides and detected by GPR40 were found to restrict autophagy and oxidative metabolism in photoreceptors [143]. The study conducted by Joyal et al. [106] also discovered that in the retinas of Vldlr^−/−^ mice with low fatty-acid uptake but high circulating lipid levels, GPR40 inhibits the expression of the glucose transporter Glut1. This inhibition results in impaired glucose entry into photoreceptors, leading to a shortage of both lipid and glucose fuels and a decrease in the levels of the Krebs cycle intermediate α-ketoglutarate (α-KG). Low levels of alpha-ketoglutarate promote the stabilization of HIF-1α and the secretion of vascular endothelial growth factor A (VEGFA) by starved Vldlr^−/−^ photoreceptors, ultimately resulting in angiogenesis (see Figure 3). In summary, GPR40 primarily regulates inflammation, endothelial dysfunction, and angiogenesis in VECs. There are fewer studies on GPR40’s role in arterial lipid metabolism and its functional regulation of VSMCs.

### 4.2. GPR43/FFAR2

GPR43 is widely expressed in adipose tissue, VECs, liver, enteroendocrine cells, pancreatic islet β-cells, and immune tissues. It plays an important role in a variety of physiological functions, as evidenced by numerous studies [144,145,146,147,148,149]. GPR43 is primarily activated by 61 SCFAs such as acetate, propionate, and butyrate [150,151]. Studies that employ the [Ca^2+^]i assay to screen for biologically active compounds as ligands have demonstrated that GPR43 can be activated by acetate and other SCFAs, such as propionate and butyrate [152]. Activation of GPR43 by SCFAs inhibits cAMP production and induces the ERK cascade through downstream Gi/o and Gq proteins. In contrast, coupling with Gq proteins promotes elevated Ca^2+^ concentrations and induces MAPK cascades [152,153,154]. Additionally, research has demonstrated that β-arrestin-2 plays a role in GPR43-dependent signalling [155]. GPR43 also activates ERK1/2 phosphorylation, which in turn promotes MAPK activation and increases Ca^2+^ concentration [148]. This was demonstrated using a bioluminescence-resonance energy-transfer approach in HEK293 cells co-transfected with eYFP-tagged GPR43 and β-arrestin-2-Renilla luciferase. The results showed that the activation of GPR43 leads to the recruitment of its downstream β-arrestin-2 [155], which inhibits nuclear translocation and promotes the expression of the pro-inflammatory NF-κB [156].

At physiological concentrations (0.1–3 mM), SCFAs can affect leukocytes and endothelial cells by activating GPR41 and GPR43 and inhibiting histone deacetylase (HDAC). SCFAs modulate various leukocyte functions, including the production of inflammatory factors (e.g., TNF-α, IL-2, IL-6, and IL-10), eicosanoids, and chemokines (e.g., MCP-1 and CINC2) [157]. Activation of GPR41/43 is responsible for the effects of acetate on IL-6 and IL-8 production, as well as the effects of butyrate and propionate on IL-6 production. Moreover, inhibition of HDACs mediates the effects of butyrate and propionate on IL-8 production, VCAM-1 expression, and PBMC adhesion to an endothelial monolayer. These findings indicate that SCFAs may play a beneficial role in preventing vascular inflammation and relevant diseases. This is achieved by the activation of GPR41/43 and the inhibition of HDACs [158]. A further study discovered that SCFAs (5–10 mM, comprising acetic acid and butyrate) enhanced Ang II-induced endothelial dysfunction by augmenting endothelial NO bioavailability. This process was associated with butyrate-mediated activation of GPR41/43, whereas acetic acid was not linked to GPR41/43 [159]. Lower levels of the human metabolite-sensitive receptor GPR41/GPR43 have been associated with arterial stiffness, which reduces its response to blood pressure-lowering metabolites, such as butyrate [160] (see Figure 3). This suggests that GPR43 regulates arterial function by affecting the inflammatory process in VECs and modulating Ang II secretion in VSMCs. However, the precise mechanism by which GPR43 regulates arterial function and its role in atherosclerosis remain to be elucidated through further experimentation.

### 4.3. GPR41/FFAR3

GPR41 is expressed in a wide range of various tissues throughout the body, including pancreatic β-cells, enteroendocrine cells, the peripheral nervous system, immune cells, and the cardiovascular system. It can regulate whole-body energetic homeostasis through SCFA-induced signalling activation [39,161,162]. GPR41 is primarily activated by propionic, butyric, and valeric acids, which are produced by the fermentation of dietary fibre bacteria in the colon [152,163]. In comparison to GPR43, GPR41 is more readily activated by longer-chain SCFAs such as valerate and hexanoate [152]. GPR41 can couple with Gi/o to inhibit cAMP production and promote phosphorylation of ERK1/2 and activation of the βγ subunit [164,165]. GPR41 also appears to couple with Gq/11 proteins, inducing a phosphatidylinositol hydrolysis cascade reaction and stimulating intracellular Ca^2+^ signalling [39]. Studies utilising proximity linkage analysis, bimolecular fluorescence complementation (BiFC), and fluorescence resonance energy transfer (FRET) have demonstrated that the GPR43/GPR41 heterodimer employs a distinct mechanism of action in comparison to its parental isoform. The heterodimer displays enhanced intracellular Ca^2+^ signalling and augmented recruitment of β-arrestin-2 yet exhibits diminished capacity to suppress cAMP production [166].

GPR41 is expressed in several peripheral arteries, and its expression is increased following arterial injury. The inactivation of the butyrate receptor GPR41 has been demonstrated to exacerbate the development of neoplastic intimal hyperplasia following injury. Additionally, GPR41-gene deficiency is associated with delayed endothelial recovery in vivo [167]. The vasodilatory effect induced by propionate-activated GPR41 is mediated by Ca^2+^-dependent eNOS activation and endothelial NO production [168]. A scratch test revealed that treatment of HUVECs with 1-methylcyclopropane carboxylate, an agonist of GPR41, promoted cell migration and proliferation. However, no such effect was observed in VSMCs [169]. Another study found that GPR41 inhibits N-type Ca^2+^ channels via Gβγ signalling, thereby reducing catecholamine release from rat-sympathetic neurons innervating vascular smooth muscle [170]. Conversely, sodium/glucose cotransporter protein (SGLT)-2 inhibitors, such as dapagliflozin and empagliflozin, are antidiabetic/diuretic drugs that have been demonstrated to possess a multitude of beneficial cardiovascular effects. It is crucial to acknowledge that these drugs stimulate the production of ketone bodies, including β-hydroxybutyrate, in the heart and blood vessels [171,172].

A study on Olfr78 (an olfactory receptor expressed in the renal juxtaglomerular apparatus and activated by SCFAs) and GPR41 demonstrated that vascular GPR41 plays a significant role in regulating blood pressure and vascular tone, as well as in the development of hypertension [173]. In particular, Olfr78 and GPR41 were identified as being expressed in the VSMCs of small resistance vessels. Mice lacking Olfr78 and GPR41 developed hypertension following antibiotic treatment, which reduced the level of SCFAs produced by gut microbial fermentation [173]. Elevated levels of butyric acid (1.4–5.8 mmol/kg) in the colon have been demonstrated to lower arterial blood pressure through colonic vagal signalling and GPR41/43 receptor-related pathways [167]. In a study by Onyszkiewicz et al. [174], it was found that valproic acid (VA), at a physiological concentration of approximately 650 μM in the colonic content and 0.4 μM in the systemic blood, was able to produce vasodilatation and reduce blood pressure when supplemented in rats at a dose of 0.15–0.6 mmol/kg. Furthermore, it was demonstrated that colonic sources of valproic acid rapidly penetrated into tissues involved in the control of blood pressure. These findings are consistent with previous studies that have demonstrated the ability of propionate and other SCFAs to induce vasodilation in vitro, resulting in an acute hypotensive response and antihypertensive protection [175,176]. Nevertheless, the signalling mechanisms responsible for this antihypertensive effect of GPR41 remain to be elucidated. Another study found that a reduction in the availability of intestinal butyrate was linked to vascular remodelling of resistance arteries in hypertensive rats [177] (see Figure 3). In summary, while GPR41 mediates a range of signalling patterns, further research is required to gain a comprehensive understanding of the mechanisms underlying its antihypertensive and vasodilatory effects. Additionally, it is essential to examine the role of GPR41 in pathophysiological processes and ascertain whether receptor heterodimerisation is a prerequisite for its distinctive signalling or function.

### 4.4. GPR120/FFAR4

GPR120 is predominantly expressed in adipose tissue, enteroendocrine cells, liver, bone, lung, and immune cells [41,42,43]. Studies have demonstrated that GPR120 can be linked to Gq proteins. The activation of GPR120 by synthetic ligands and agonists, such as LCFAs, leads to an increase in intracellular Ca^2+^ levels without affecting cAMP concentrations [178,179]. FFAs promote glucagon-like peptide-1 (GLP-1) secretion by activating GPR120 in enteroendocrine STC-1 cells. Mice with oral ALA exhibit elevated levels of plasma insulin and GLP-1. To date, no study has demonstrated that GPR120 can be coupled to Gi or Gs proteins. Despite sharing only 10% homology in their amino-acid sequences, GPR120 and GPR40 exhibit similar ligand sensitivity and activity. Furthermore, GPR120 is more readily activated by PUFAs [180,181]. In contrast to GPR120S, which exerts its effects through the Gq/11 and β-arrestin-related pathways, GPR120L exerts its bioefficacy only through the activation of the β-arrestin-related pathway and does not cause an increase in intracellular Ca^2+^ concentration [182,183]. Additionally, GPR120L is expressed in a limited number of tissues in comparison to GPR120S, which is only detected in the colon. This is in contrast to GPR120S, which is expressed in multiple tissues [131].

With regard to arterial function, GPR120 has been demonstrated to play a protective role in the development of atherosclerosis [184]. In HUVECs and THP-1 monocytes treated with Ginsenoside Rb2, there was an increase in GPR120, AMPK phosphorylation, and HO-1 expression. Furthermore, siRNA silencing of GPR120 resulted in a reduction in Rb2-induced AMPK phosphorylation. Taken together, Rb2 has been demonstrated to reduce inflammation and endothelial stress, inhibit LPS-mediated apoptosis, and decrease the adhesion level of HUVECs through the GPR120/AMPK/HO-1-related pathway [185]. Hwang et al. [186] discovered that protectin DX (PDX) can reverse the production of ROS induced by hydrogen peroxide (H_2_O_2_), the Bax/Bcl-2 ratio, and the loss of mitochondrial membrane potential. This leads to an increase in cell viability and a decrease in the release of lactic dehydrogenase (LDH). Furthermore, PDX also enhances the expression and activity of antioxidant proteins such as catalase and superoxide dismutase 2 (SOD2). These proteins were inhibited by AMPK inhibitors and GPR120 antagonists. The evidence suggests that the PDX/AMPK axis protects VECs against OS induced by H_2_O_2_. Regarding vascular growth and migration, Zhang et al. [187] discovered that activating GPR120 in human breast cancer cells stimulated the secretion of vascular endothelial growth factor (VEGF) and IL-8, as well as cell migration and epithelial-mesenchymal transition. In HUVECs, 100 μM DHA inhibited VEGF-induced cell migration through the GPR120/PP2A/ERK1/2/eNOS signalling pathway [188].

In VSMCs, the EPA was observed to inhibit the activation of the TAK-1/JNK pathway and the expression of MMP-9. This effect was negated by the knockdown of the GPR120/AF-4 receptor. Additionally, the Trail/TAK-1/JNK/MMP-9 pathway has been demonstrated to promote the development of abdominal aortic aneurysm in OPG KO mice [189]. Another study found that EPA prevented the progression of intracranial aneurysms in rats [190]. Additionally, EPA (20–30 mg/kg) inhibits inflammatory responses induced by acute cerebral infarction by blocking NLRP3 inflammasome activation. Moreover, it attenuated apoptosis induced by acute cerebral infarction by inhibiting NLRP3 inflammatory vesicle activation via GPR40 and GPR120 [191]. In a study conducted by Nakamura et al. [192], the preventive effects of EPA on arterial calcification were confirmed. The study also found that EPA reduces NOX gene expression and activity via GPR120 in klotho mutant mice. In atherosclerosis-related studies, there was a negative correlation between AMPKα2 levels and UBC9 expression in human arteries. In VSMCs, the inactivation of AMPKα2 resulted in increased SUMOylation of the fatty-acid receptor GPR120, which blocked fish oil-induced internalisation and binding to β-arrestin. This suggests that AMPKα2 controls the anti-atherosclerotic effect of fish oil by regulating SUMOylation of GPR120 [193] (see Figure 3). In conclusion, GPR120 regulates the processes of inflammation, lipid metabolism, endothelial adhesion, and migration of VSMCs in VECs and plays a crucial role in angiogenic migration and protection against atherosclerosis and arterial calcification.

In summary, studies investigating the regulation of arterial function by FFARs are currently focused on in vitro cellular experiments. The majority of these studies investigate the beneficial effects of supplementation with SCFAs and UFAs on arterial function through the modulation of FFARs. Limited animal experiments have also indicated that the absence of FFARs is an important reason for the occurrence of arterial dysfunction. To date, studies have not been able to confirm that the deleterious effects of high concentrations of FFAs in the body on arteries are mediated through FFARs.

## 5. Conclusions

An excess of FFAs is a significant contributor to dyslipidaemia in the body. They affect the secretion of adhesion molecules, cytokines, and inflammatory factors in VECs, cholesterol transport, and promote OS levels. This leads to the development of endothelial inflammation, endothelial cell apoptosis, endothelial oxidative damage, and endothelial dysfunction. FFAs have been demonstrated to stimulate the proliferation, migration, and apoptosis of VSMCs, as well as the accumulation of lipids in VSMCs. This results in vascular hypertrophy and vessel-wall stiffness, which are associated with the development of arterial hypertension and atherosclerosis. These factors are significant contributors to arterial dysfunction. However, studies have indicated that FFARs may be implicated in arterial function-related processes, including the proliferation, differentiation, migration, apoptosis, inflammation, and angiogenesis of VECs and VSMCs. The FFARs play a multifaceted role in the pathogenesis of hypertension, endothelial dysfunction, and atherosclerosis. From the above, it can be concluded that the regulatory effects of excess FFAs on arterial function are predominantly negative, whereas FFARs have a mostly positive effect on arterial function. It is evident that this phenomenon is in opposition to the detrimental effects of elevated concentrations of FFAs on arteries. For example, high levels of PA in the organism adversely affect arterial function, whereas activation of FFARs by high levels of PA positively affects arteries. However, the current evidence does not yet confirm whether the deleterious effects of elevated concentrations of FFAs on arteries are related to their binding to FFARs.

Consequently, the overlapping signalling patterns between FFAs and FFARs, as well as the relationship between these patterns and arterial function, tissue expression patterns, and function, represent a significant challenge to the characterisation of their physiology and pharmacology. The role of FFAs and FFARs in cardiovascular homeostasis is regulatory and adjunctive rather than causal or absolutely essential. However, they play a significant role in regulating biological processes in response to changes in nutritional status and in linking dietary effects to arterial function or CVDs. The regulation of arterial function by FFARs is a relatively new field of study. Although it is known that FFARs can regulate arterial function, the specific direction and molecular mechanisms of this regulation by different receptors remain unclear. Therefore, in the future, this field should aim to validate and explain the specific mechanism by which FFAs regulate arterial function through the activation of FFARs. It is important to provide a scientific explanation of the role of FFARs in regulating arterial function at the molecular level, which will serve as a reliable basis for the development of drugs for arterial dysfunction.

## Figures and Tables

**Table 1:** Classification and Production Pathways of FAs.

FAs	Systematic Name	Common Name	Number of Carbon Atoms	Formula	Pathways/Reactions	Source/Producers	References
SCFAs	Methanoic acid	Formic acid	1	HCOOH	Disposal via Catalase and Folate-Dependent Mechanisms	*Fusobacterium nucleatum*	[52,53]
	Ethanoic acid	Acetic acid	2	CH_3_COOH	Pyruvate via acetyl-CoA	The enteric bacteria; *Akkermansia muciniphilia* (Representative of species bacteria); *Bateroides* spp.; *Bifidobacterium* spp.; *Prevotella* spp.; *Ruminococcus* spp.	[54,55]
					Wood-Ljungdahl pathway	*Blautia hydrogentrophica*, *Chrostridium* spp.; *Streptococcus* spp.	
	Propanoic acid	Propionic acid	3	CH_3_CH_2_COOH	Succinate pathway	Bacteroides spp.; Phascolarctobacterium succinatutens, Dalister spp.; Veilonella spp.	[54,56]
					Acrylate pathway	Megasphaera elsdenii, Coprpcoccus catus	
					Propanediol pathway	*Salmonella* spp.; *Roseburia inulinivorans*; *Ruminocossus obeum*	
	Butanoic acid	Butyric acid	4	CH_3_(CH_2_)_2_COOH	Phosphotransbutyrylase/Butyrate kinase route	Coprococcus comes; *Coprococcus eutactus*	[54,57]
					Butyryl-CoA: acetate CoA-transferase route	*Anaerostripes* spp. (A, L); *Coprococcus catus* (A); *Eubacterium rectale* (A); *Eubacterium hallii* (A, L); *Faecalibacterium prausnitzii* (A); *Roseburia* spp. (A)	
	Pentanoic acid	Valeric acid	5	CH_3_(CH_2_)_3_COOH		*Oscillibacter valericigenes*; *Megasphaera elsdenii*	[58,59]
MCFAs	Octanoic acid	Caprylic acid	8	CH_3_(CH_2_)_6_COOH	Fatty acid β-oxidation	Absorbed from dietary plant oils and milk directly into the portal blood, e.g., dairy products\palm kernel, coconut oils	[60,61,62]
	Decanoic acid	Capric acid	10	CH_3_(CH_2_)_8_COOH			
	Dodecanoic acid	Lauric acid	12	CH_3_(CH2)_10_COOH			
LCFAs	Tetradeconic acid	Myristic acid	14	CH_3_(CH_2_)_12_COOH	Fatty acid β-oxidation	Diet, e.g., olive oil.	[63,64]
	Hexadecanoic acid	Palmitic acid	16	CH_3_(CH_2_)_14_COOH			
	Octadecanoic acid	Stearic acid	18	CH_3_(CH_2_)_16_COOH			

## Data Availability

No data was used for the research described in the article.

## Abbreviations

AC	adenylate cyclase
Akt	serine/threonine kinase
AMPK	AMP-activated protein kinase
Ang II	angiotensin II
cAMP	cyclic adenosine monophosphate
[Ca^2+^]i	intracellular free Ca^2+^ concentration
CAPE	caffeic acid phenethyl ester
CCL2	chemokine (C-C motif) ligand 2
CCL4	chemokine (C-C motif) ligand 4
cGAS	cyclic bird adenylate synthetase
CINC2	cytokine-induced neutrophil chemoattractant 2
COX-2	cyclooxygenase-2
CREB	cAMP-response element binding protein
CVDs	cardiovascular diseases
Cx43	gap connexin 43
DAMPs	damage-associated molecular patterns
DHA	docosahexaenoic acid
ECM	extracellular matrix
eNOS	endothelial nitric oxide synthase
EPA	eicosapentaenoic acid
ERK	extracellular signal-regulated kinase
ERK1/2	extracellular regulatory protein kinase1/2
FFARs	free fatty acid receptors
FFAs	free fatty acids
GLP-1	glucagon-like peptide-1
GPCRs	G protein-coupled receptors
H_2_O_2_	hydrogen peroxide
HDAC	histone deacetylase
HDAC	histone deacetylase
HIF-1α	hypoxia-inducing factor 1α
HO-1	heme oxygenase-1
HUVSMCs	human umbilical vein smooth muscle cells
ICAM-1	intercellular adhesion molecule-1
IKK	I-kappa-B kinase
IL	interleukin
IP3	inositol triphosphate
IR	insulin resistance
IRF3	interferon regulatory factor 3
JNK	c-Jun amino-terminal kinase
KLF2	Kruppel-like factor 2
LA	linoleic acid
LAE	large arterial elasticity
LCFAs	long-chain fatty acids
LDH	lactic dehydrogenase
LPS	lipopolysaccharide
MAPK	mitogen-activated protein kinase
MCFAs	medium-chain fatty acids
MCP-1	monocyte chemoattractant protein-1
MIP-1α	macrophage inflammatory protein-1α
miRs	microRNAs
MMP-9	matrix metalloproteinase 9
MUFAs	monounsaturated fatty acids
NADPH	nicotinamide adenine dinucleotide phosphate
NEFAs	non-esterified fatty acids
NF-κB	nuclear factor kappa B
NO	nitric oxide
NRF2	nuclear factor erythroid 2-related factor 2HO-1
OA	oleic acid
OS	oxidative stress
ox-LDL	low-density lipoprotein
PA	palmitic acid
PCNA	proliferating cell nuclear antigen
PGC1	peroxisome bioactivator receptor gamma coactivator 1
PI3K	phosphatidylinositol-3-hydroxykinase
PKC	protein kinase C
PLC	phospholipase C
PP2Cb	protein phosphatase 2Cb
PPARγ	peroxisome proliferator-activated receptor γ
PUFAs	polyunsaturated fatty acids
RAS	renin-angiotensin system
RGZ	Rosiglitazone
ROS	reactive oxygen species
RPE	retinal pigment epithelial
SAE	small arterial elasticity
SCFAs	short-chain fatty acids
SFAs	saturated fatty acids
SOD2	superoxide dismutase 2
SOX2	sex determination region Y box protein 2
STING	stimulator of interferon genes
TAK-1	transforming growth factor-beta activating protein 1
TLR-2	Toll-like receptor-2
TLR-4	Toll-like receptor-4
TNF-α	tumor necrosis factor α
TNF-β	tumor necrosis factor β
UFAs	unsaturated fatty acids
VA	valproic acid
VCAM-1	vascular cell adhesion molecule-1
VECs	vascular endothelial cells
VEGF	vascular endothelial growth factor
VEGFA	vascular endothelial growth factor A
VEGFR	vascular endothelial growth factor receptor
VSMCs	vascular smooth muscle cell
YAP	Yes-associated protein
α-KG	α-ketoglutarate
